# Supplementary material for: Galectin-4 Is Involved in the Structural Changes of Glycosphingolipid Glycans in Poorly Differentiated Gastric Cancer Cells with High Metastatic Potential
Source: Int J Mol Sci. 2023 Aug 1;24(15):12305. doi: 10.3390/ijms241512305 (PMC10418866; doi:10.3390/ijms241512305)
Supplement: Supplementary file 1 [file ijms-24-12305-s001.zip › ijms-2506456-supplementary.pdf]

Supplemental Materials

Table S1. The list of abbreviations of *N*-glycans from NUGC4 cells.

m/z (theoretical) of BOA-glycan (Na+)	glycan composition	abbreviation
1022.380	H2N2F1	M2F
1184.433	H3N2F1	M3F
1200.428	H4N2	M4
1362.481	H5N2	M5
1524.534	H6N2	M6
1686.587	H7N2	M7
1848.639	H8N2	M8
2010.692	H9N2	M9
2117.777	H5N5F1	G2F+GN
2263.835	H5N5F2	G2F2+GN
2336.851	H6N6	G3+GN
2409.893	H5N5F3	G2F3+GN
2482.909	H6N6F1	G3F+GN
2571.946	H6N5F3	G3F3
2628.967	H6N6F2	G3F2+GN
2701.984	H7N7	G4+GN
2775.025	H6N6F3	G3F3+GN
2848.041	H7N7F1	G4F+GN
2921.083	H6N6F4	G3F4+GN
2994.099	H7N7F2	G4F2+GN
3140.157	H7N7F3	G4F3+GN
3286.215	H7N7F4	G4F4+GN
3432.273	H7N7F5	G4F5+GN
3505.289	H8N8F3	G5F3+GN
3651.347	H8N8F4	G5F4+GN
3797.405	H8N8F5	G5F5+GN

The left column shows the *m/z* of BOA-labeled *N*-glycans (Na+), glycan composition (H, hexose; N, *N*-acetylhexosamine; F, fucose), and the abbreviations of *N*-glycans.

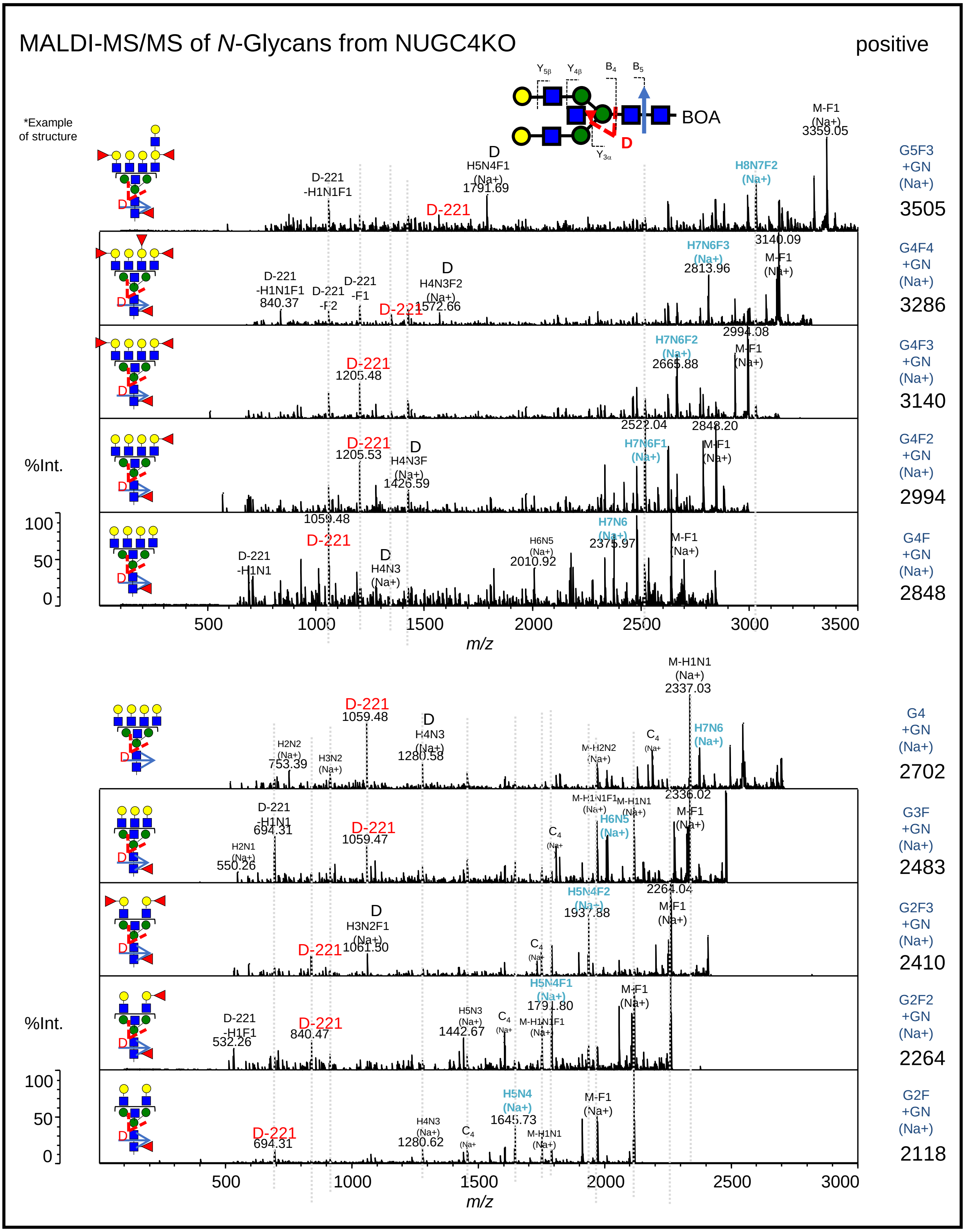

Figure S1. Detailed structure of *N*-glycans in NUGC4 cells identified by MALDI-MS^2. MS^2 sequencing at *m/z* 2118, 2264, 2410, 2483, 2702, 2848, 2994, 3140, 3286, and 3505 of Fig.1 were acquired in positive ion mode. The presence of bisecting GlcNAc was identified by the fragment ion peak of D ion-221 (bisect structural marker) [18, 19]. The compositions and structures of *N*-glycans are shown in the MS spectra.

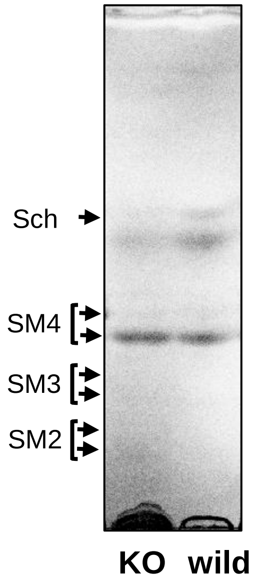

Figure S2. HPTLC analysis of acidic lipids from NUGC4 cells. Acidic lipids, containing sulfated glycosphingolipids (GSLs) are indicated by their abbreviations: galactosylceramide sulfate (SM4), lactosylceramide sulfate (SM3), gangliotriaosylceramide monosulfate (SM2), and cholesterol 3-sulfate (Sch). The TLC bands of sulfated lipids were visualized using Azure A staining.

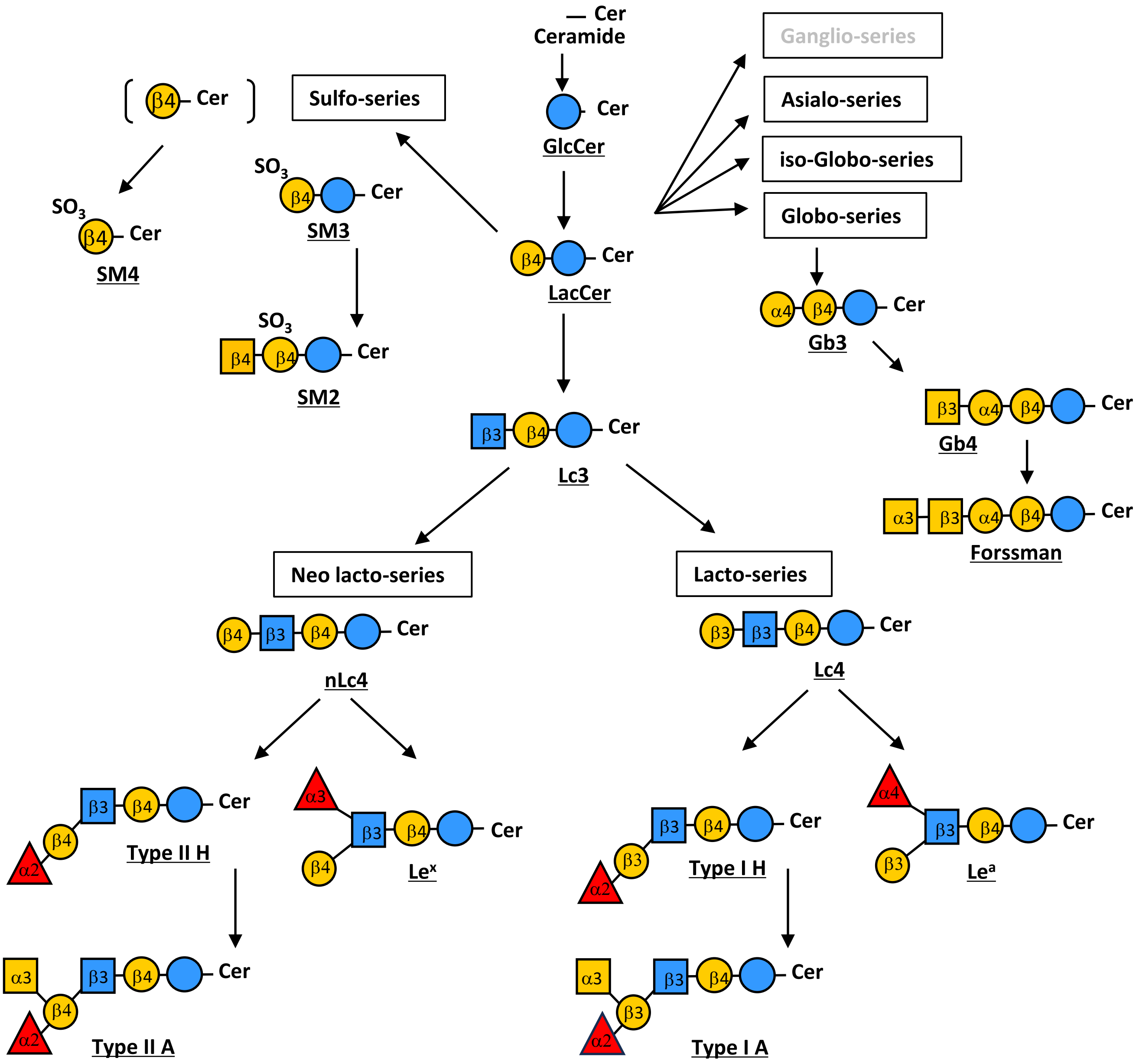

Figure S3. Possible GSL biosynthetic pathways in NUGC4 cells. The GSLs are indicated by their abbreviations.

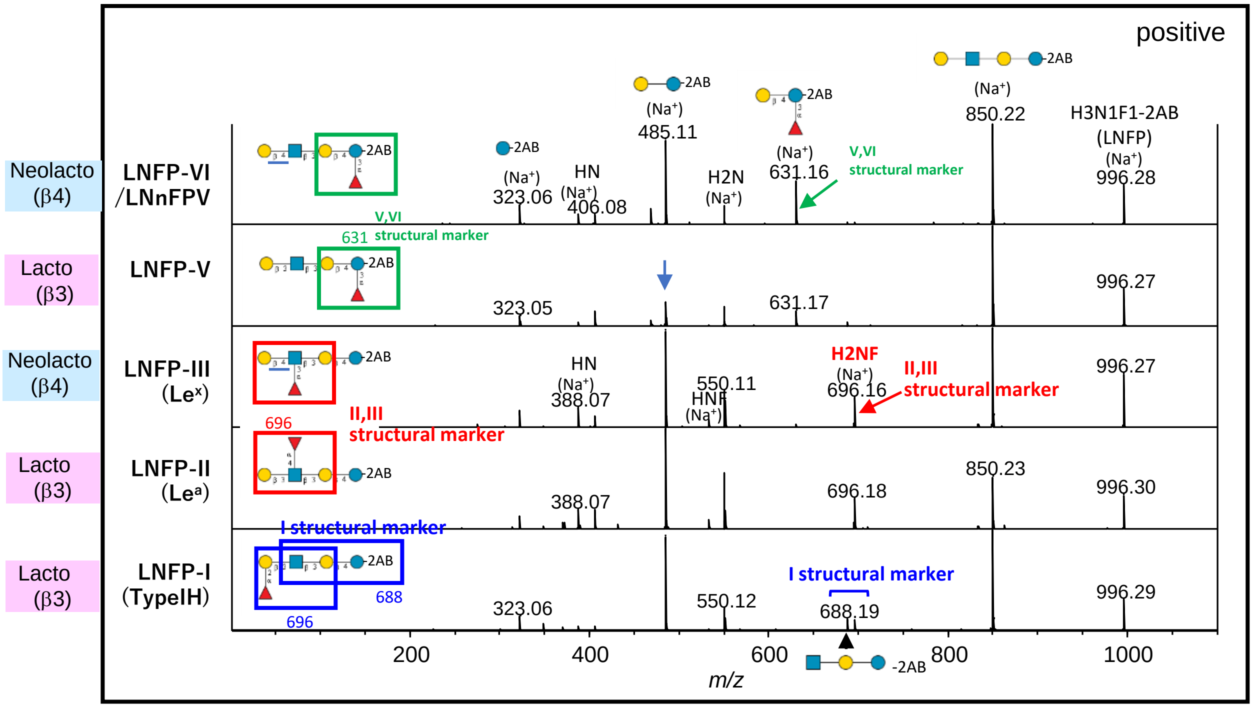

Figure S4. MALDI-MS^n analysis of five standards (STDs) of H3N1F1 (Lacto-N-fucopentaose, LNFP I–III, and V–VI) with 2-aminobenzamide (2AB) labeling. Reference MALDI-MS^2 spectra for matching with NUGC4 samples were acquired in positive ion mode. LNFP I (Type IH, Lc-type), LNFP II (Le^a, Lc-type), LNFP III (Le^x, nLc-type), LNFP V (Lc-type), and LNFP VI/LNnFP V (nLc-type). The structures of LNFP I-VI and structural markers of the fragment peaks are shown in the MS^2 spectra.

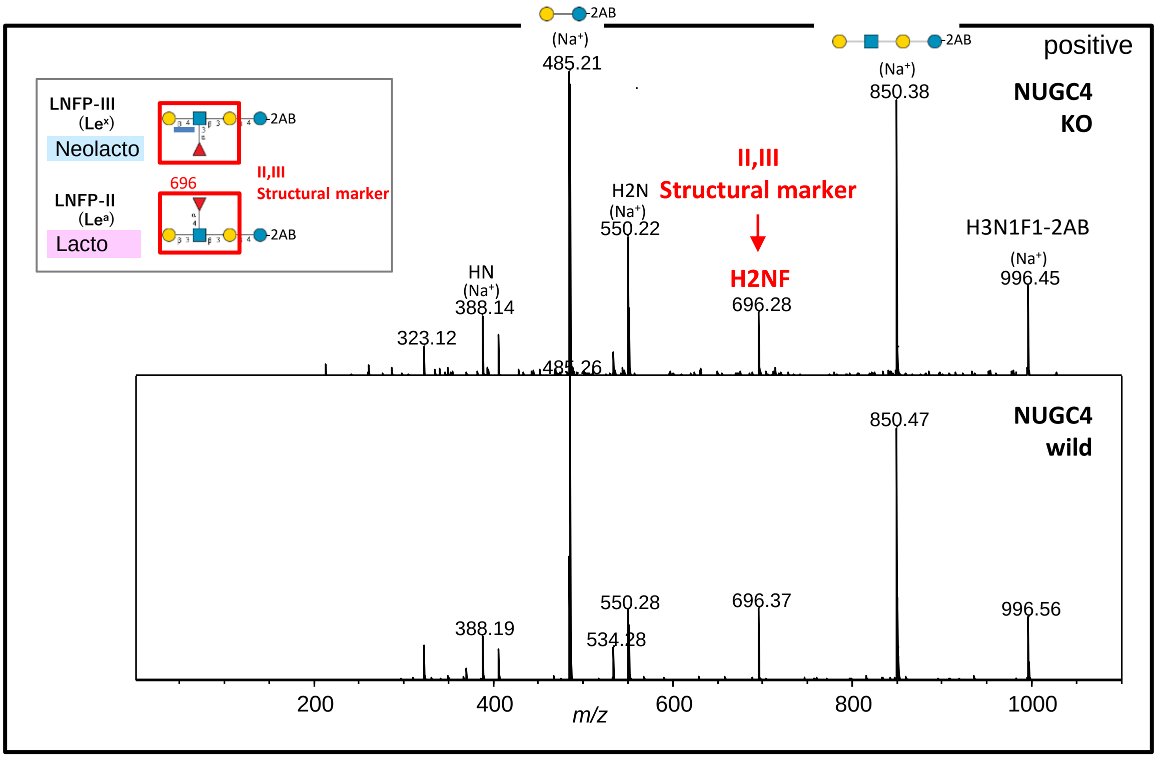

Figure S5. MALDI-MS^2 analysis of the GSL glycans, H3N1F1 (*m/z* 996), from the wild-type NUGC4 (lower) and galectin-4 KO (upper) with 2AB-labeling. MALDI-MS^2 was acquired in positive ion mode. The structures of LNFP II and III and the structural markers of the fragment peaks are shown in the MS^2 spectra.

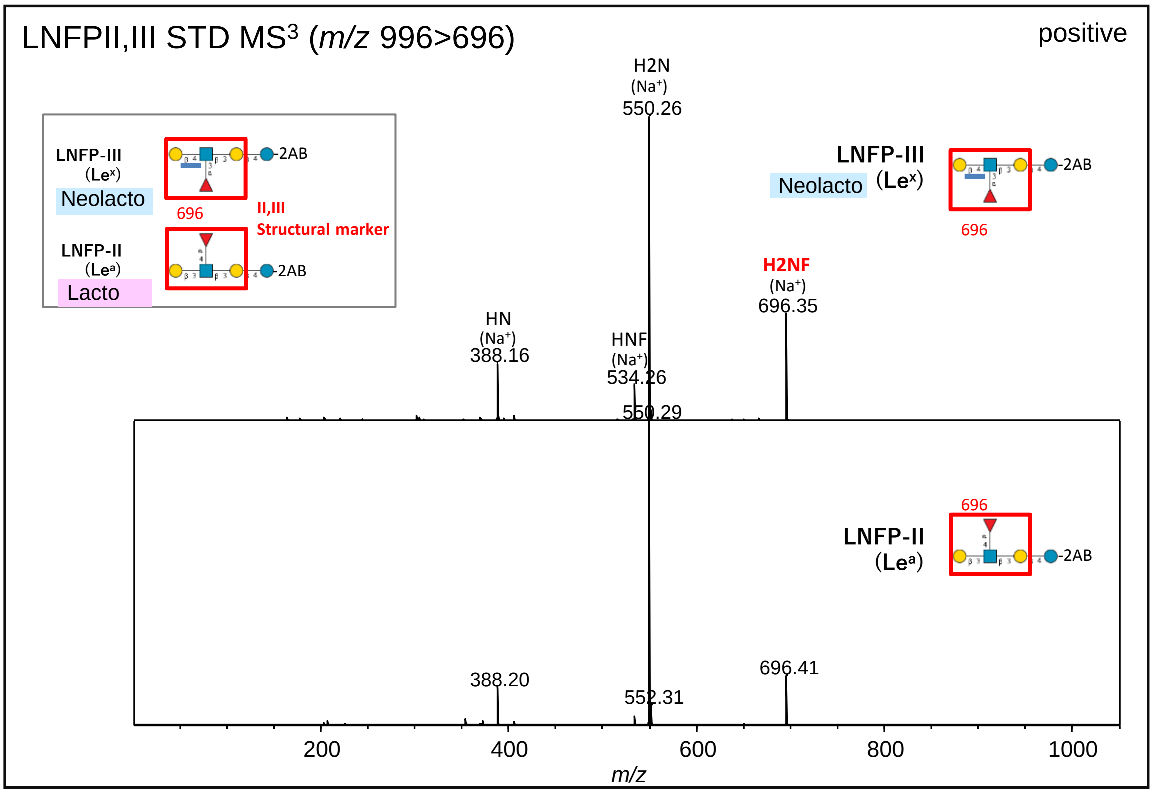

Figure S6. MALDI-MS^3 analysis (*m/z* 996 > 696) of the STDs of LNFP II (lower) and LNFP III (upper) with 2AB-labeling. MALDI-MS^3 spectra were acquired in positive ion mode. The structures of LNFP II and III and the structural markers of the fragment peaks are shown in the MS^2 spectra.

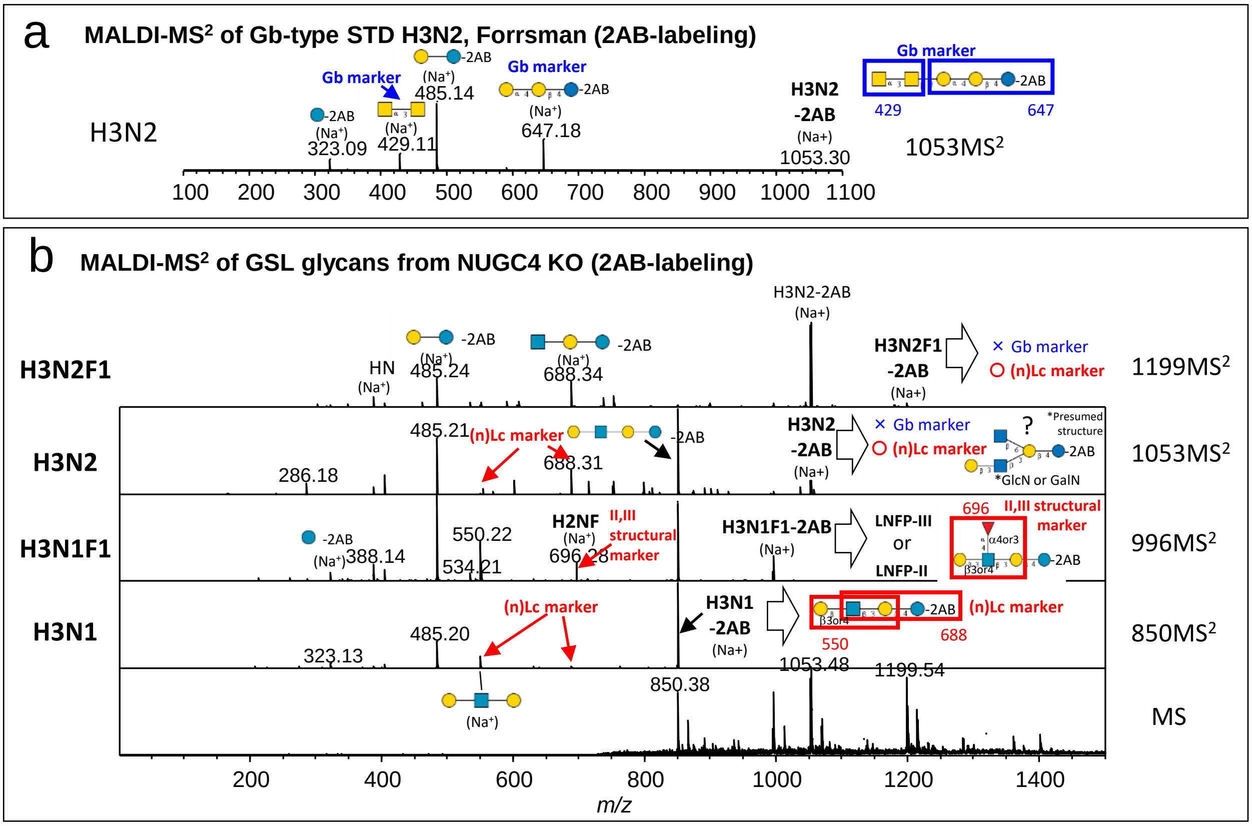

Figure S7. (a) Reference MALDI-MS² data for STD H3N2 (Forssman), a globo (Gb)-type glycan, with 2AB-labeling. MALDI-MS² spectra were acquired in positive ion mode. The structures and structural markers (Gb markers) of the fragment peaks are shown in the MS² spectra. (b) MALDI-MS² analysis of GSL glycans H3N1, H3N1F1, H3N2, and H3N2F1 from NUGC4 galectin-4 KO with 2AB labeling. MALDI-MS² spectra were acquired in positive ion mode. Based on the MS data, MS² data of H3N1 (*m/z* 850), H3N1F1 (*m/z* 996), H3N2 (*m/z* 1053), and H3N2F1(*m/z* 1199) were obtained. The composition, presumed structure, and structural markers (Lacto/neoLacto, (n)Lc marker) of the fragment peaks are shown in the MS² spectra.

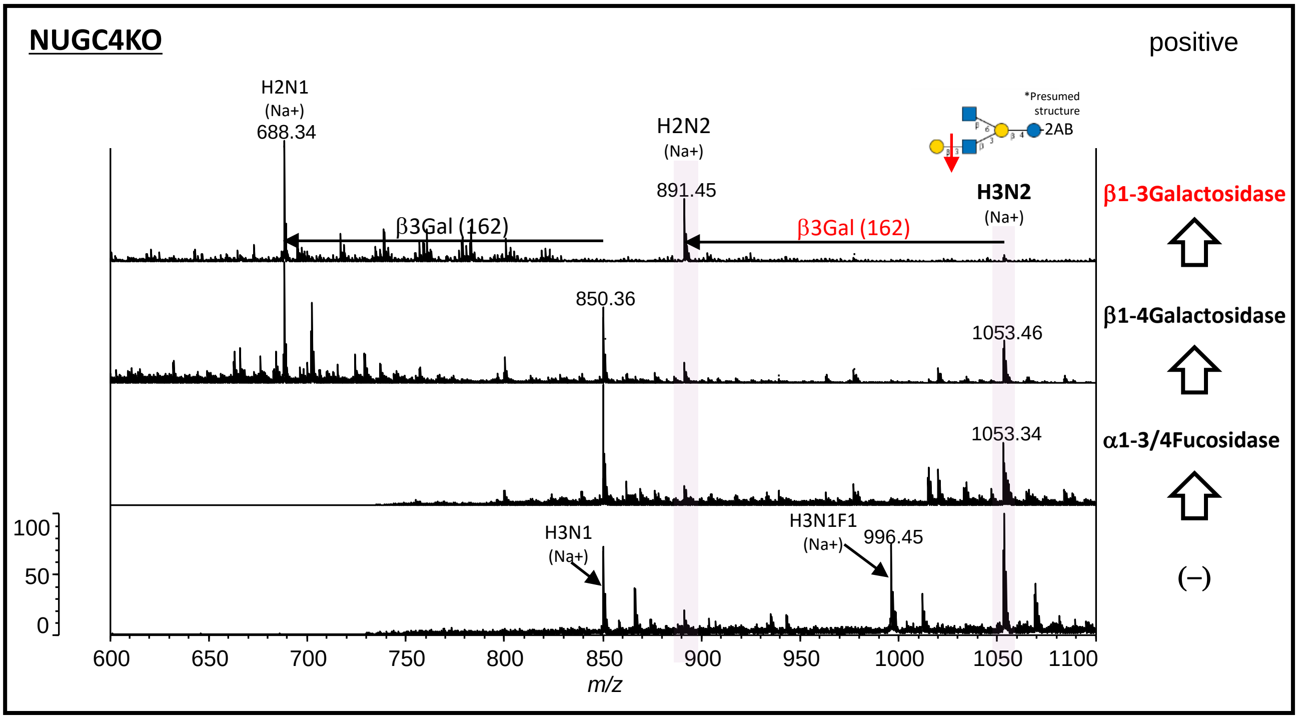

Figure S8. The detailed structure of the GSL glycan H3N2 (*m/z* 1053) in NUGC4 galectin-4 KO cells was identified using glycosidase and MS analysis. From the bottom, the MALDI-TOF MS spectra of GSL glycans without glycosidase, with α1-3/4Fucosidase, with β1-4Galactosidase after α1-3/4Fucosidase, with β1-3Galactosidase after β1-4Galactosidase, and α1-3/4Fucosidase. MALDI-TOF MS spectra of GSL glycans with 2AB-labeling were acquired in positive ion mode. The compositions and presumed structures of the glycans are shown in the MS spectra.

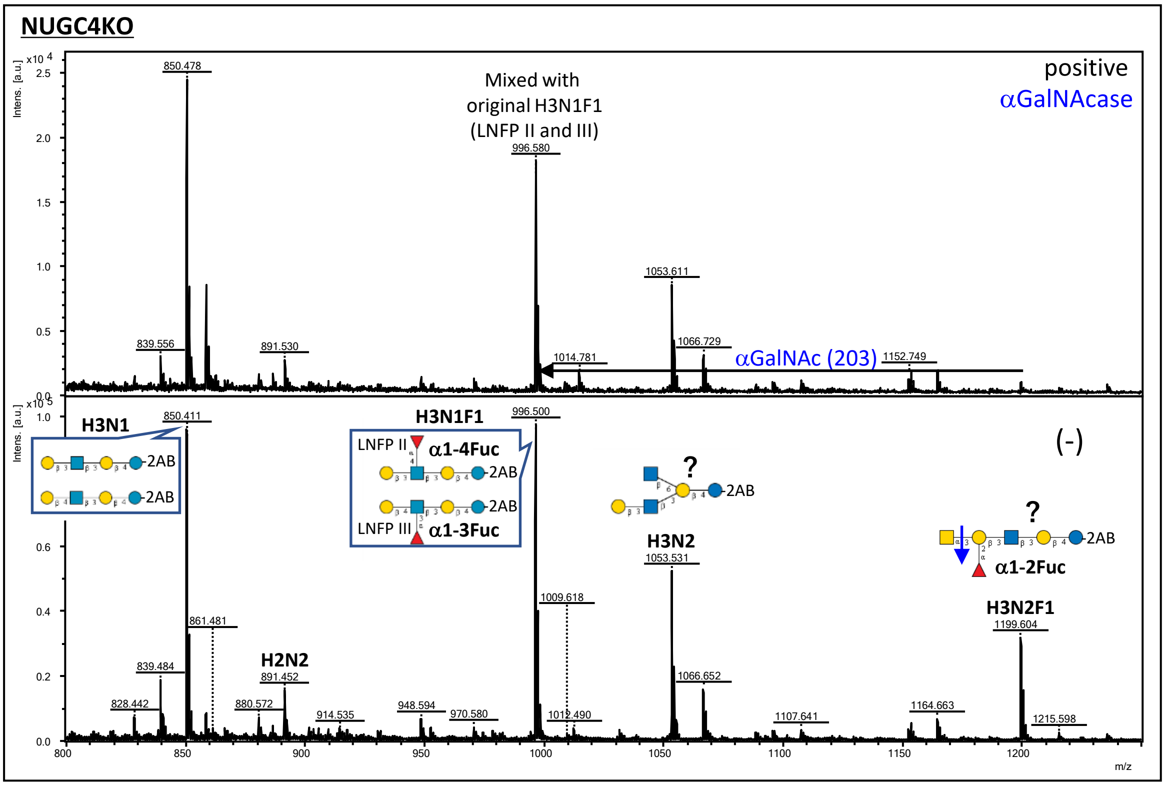

Figure S9. The detailed structure of the GSL glycan H3N2F1 (*m/z* 1199) in NUGC4 galectin-4 KO cells was identified using glycosidase and MS analysis. The MALDI-TOF MS spectra of 2AB-labeled GSL glycans without (lower) and with α-*N*-acetyl-galactosaminidase (αGalNAcase, upper) were acquired in positive ion mode. The compositions and presumed structures of the glycans are shown in the MS spectra.

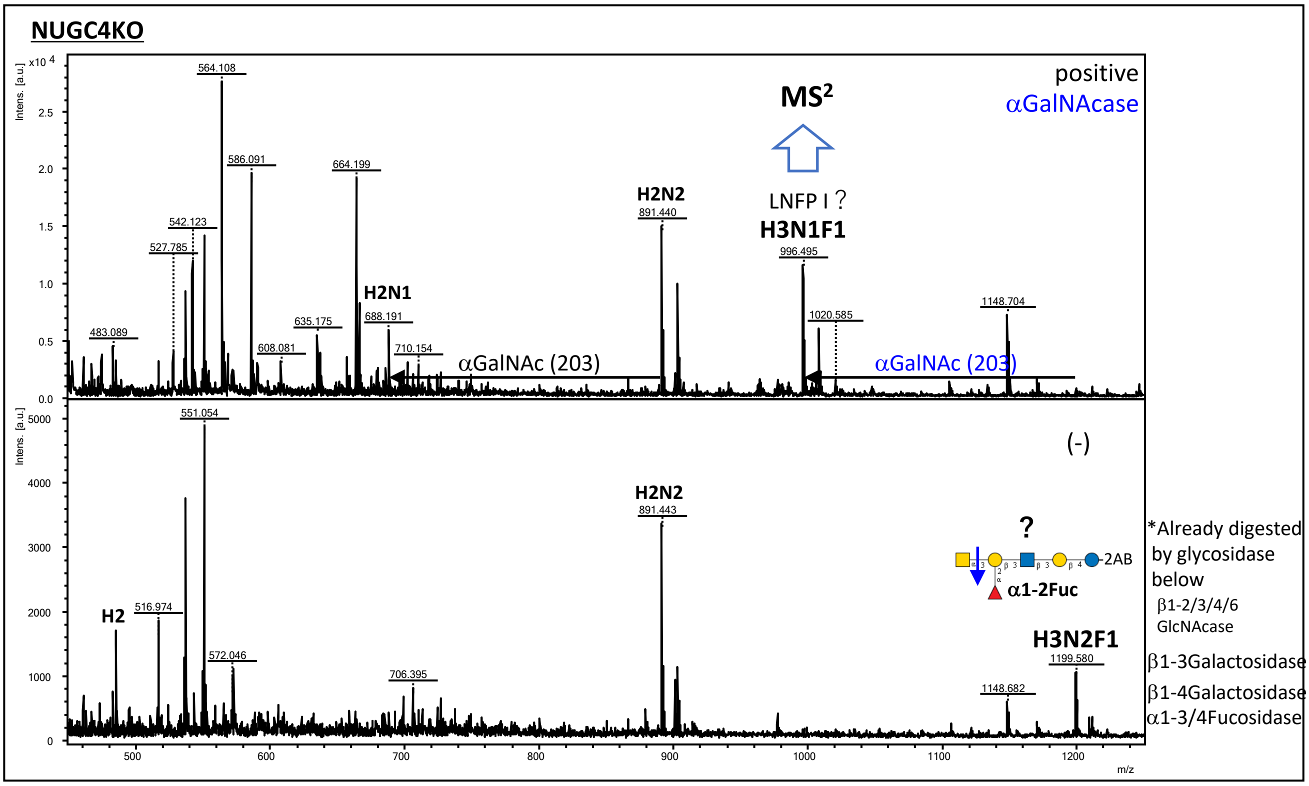

Figure S10. The detailed structure of the GSL glycan H3N2F1 (*m/z* 1199) in NUGC4 KO cells was identified using glycosidase and MS analysis. MALDI-TOF MS spectra of glycans digested with (upper) or without (lower) αGalNAcase. Glycan was digested with β-*N*-acetyl-glucosaminidase, β1-3Galactosidase, β1-4Galactosidase, and α1-3/4Fucosidase prior to αGalNAcase digestion. MALDI-TOF MS spectra were acquired in positive ion mode. The compositions and presumed structures of the glycans are shown in the MS spectra.

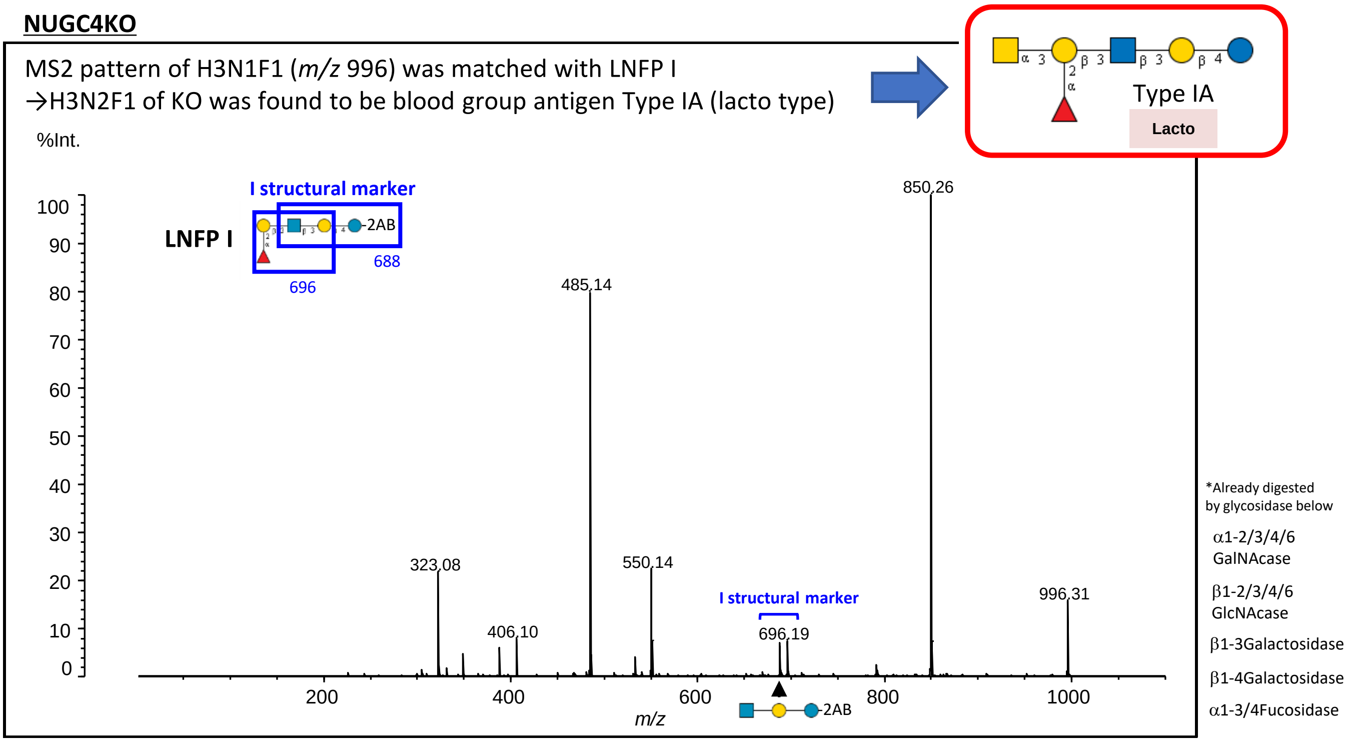

Figure S11. MALDI-MS^2 analysis of the GSL glycan, H3N1F1 (*m/z* 996), was digested with αGalNAcase in the NUGC4 KO with 2AB-labeling. MALDI-MS^2 was acquired in positive ion mode. The GSL glycan structures and structural markers of the fragment peaks are shown in the MS^2 spectra.

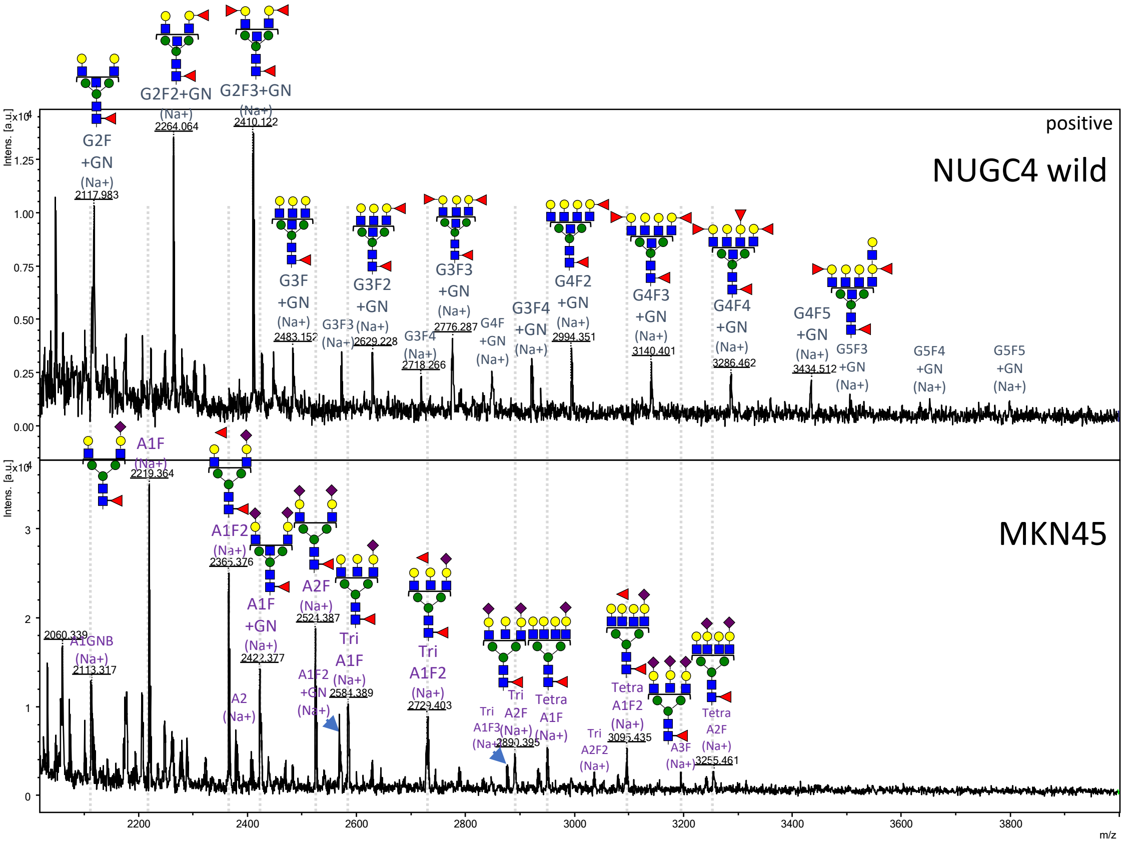

Figure S12. Analysis of the *N*-glycans of the membrane protein from NUGC4 and MKN45 cells by MALDI-TOF MS (BOA-labeling). MALDI-TOF MS spectra (m/z 2020 to 4000) in wild-type NUGC4 (upper) and MKN45 (lower), were acquired in positive ion mode.
